# Poly(A)-Binding Protein Cytoplasmic 1 Inhibits Porcine Epidemic Diarrhea Virus Replication by Interacting with Nucleocapsid Protein

**DOI:** 10.3390/v14061196

**Published:** 2022-05-31

**Authors:** Tingting Wu, Xiaona Wei, Shumei Zheng, Gaoli She, Zhenling Han, Zhichao Xu, Yongchang Cao, Chunyi Xue

**Affiliations:** 1 State Key Laboratory of Biocontrol, Life Sciences School, Sun Yat-sen University, Guangzhou 510275, China; wutt8@mail2.sysu.edu.cn (T.W.); weixn6@mail.sysu.edu.cn (X.W.); zhengshm9@mail2.sysu.edu.cn (S.Z.); shegaoli@gdph.org.cn (G.S.); hanzhling@mail2.sysu.edu.cn (Z.H.); xuzhich5@mail.sysu.edu.cn (Z.X.); caoych@mail.sysu.edu.cn (Y.C.); 2 Wen’s Group Academy, Wen’s Foodstuffs Group Co., Ltd., Yunfu 527400, China

**Keywords:** porcine epidemic diarrhea virus, poly(A)-binding protein cytoplasmic 1, antiviral effect, nucleocapsid protein

## Abstract

Porcine epidemic diarrhea virus (PEDV) is the etiological agent of porcine epidemic diarrhea (PED) characterized by vomit, watery diarrhea, dehydration and high mortality. Outbreaks of highly pathogenic variant strains of PEDV have resulted in extreme economic losses to the swine industry all over the world. The study of host–virus interaction can help to better understand the viral pathogenicity. Many studies have shown that poly(A)-binding proteins are involved in the replication process of various viruses. Here, we found that the infection of PEDV downregulated the expression of poly(A)-binding protein cytoplasmic 1 (PABPC1) at the later infection stage in Vero cells. The overexpression of PABPC1 inhibited the proliferation of PEDV at transcription and translation level, and siRNA-mediated depletion of PABPC1 promoted the replication of PEDV. Furthermore, mass spectrometry analysis and immunoprecipitation assay confirmed that PABPC1 interacted with the nucleocapsid (N) protein of PEDV. Confocal microscopy revealed the co-localizations of PABPC1 with N protein in the cytoplasm. Taken together, these results demonstrate the antiviral effect of PABPC1 against PEDV replication by interacting with N protein, which increases understanding of the interaction between PEDV and host.

## 1. Introduction

Porcine epidemic diarrhea virus (PEDV), a member of the genus *Alphacornavirus*, mainly infects suckling piglets and causes porcine epidemic diarrhea (PED) characterized by vomit, watery diarrhea, dehydration, and high mortality [1,2,3,4]. PED was first reported in England in 1971 and spread to other swine production countries subsequently [5]. Since 2010, outbreaks of PED have resulted in extreme economic losses to the swine industry all over the world because of the emergence of highly pathogenic mutant strains [4,6]. PEDV is a positive-sense single-strand RNA virus and the whole genome is approximately 28 kb including the 5′-untranslated region (UTR), open reading frame (ORF) 1a/1b, spike (*S*), ORF3, envelope (*E*), membrane (*M*), nucleocapsid (*N*) genes, and 3′-UTR. Poly(A) tail is necessary during coronavirus genome replication [7]. The whole genome of PEDV translates into four structural proteins, S, E, M and N, and 16 nonstructural proteins [8]. As a major component of the nucleocapsid structure, N protein has a variety of biological functions [9]. By activating nuclear factor kappa-light-chain-enhancer of activated B cells and upregulating interleukin-8 expression, N protein antagonizes interferon (IFN) production and disrupts the antiviral response of host cells [10,11]. N protein has good immunogenicity and can induce a strong cellular immune response [12].

The study of host antiviral factors can help to better understand the host–virus interaction. Several antiviral factors have been reported to show antiviral activity against PEDV infection. Bone marrow stromal cell antigen 2 suppresses PEDV replication by targeting and degrading N protein with selective autophagy [12]. Transferrin receptor 1 levels at the cell surface influence the susceptibility of newborn piglets to PEDV infection [13]. Tomatidine inhibits PEDV replication by targeting 3CL protease [14]. Viperin interacts with the viral N protein to inhibit PEDV proliferation [15]. Moreover, cholesterol 25-hydroxylase, GTPase-activating protein-binding protein 1, interleukin-11 and IFN-λ can regulate PEDV infection and replication [16,17,18,19].

Poly(A)-binding protein cytoplasmic 1 (PABPC1), one of the poly(A)-binding proteins (PABPs), is composed of four non-identical RNA-recognition motifs (RRMs) and a C-terminus which consists of a proline-rich region and a globular domain [20]. In the cytoplasm, PABP binds to the poly(A) tail at the 3‘end of mRNA through RRMs and interacts with the N terminus of eukaryotic translation initiation factor 4 gamma (eIF4G) protein. The interaction of PABP, mRNA and eIF4G constitutes a translation initiation complex, which mediates cellular mRNA circularization and enhances cap-dependent translation by facilitating ribosome recycling [21,22]. PABP can also interact with deadenylated protein complex to promote the degradation of mRNA [20]. Many RNA and DNA viruses inhibit the translation of host cells as a means of interfering with the cell defense system. The NSP3A protein of rotavirus can bind to eIF4G to transfer PABPC1 from the translation complex [23]. The 2A and 3C proteases of picornavirus can inactivate PABPC1 by cleaving the N-terminal of PABPC1 so that it cannot bind to eIF4G, thus affecting the normal translation of the host [24,25,26]. In addition, PABPC1 can promote or inhibit the translation of virus mRNA in various pathways. For example, in the absence of a poly(A) tail, PABP binds to the 3′-UTR of Dengue virus to promote translation [27]. However, PABPC4 inhibits PEDV replication by degrading the N protein [28].

In our previous studies about transcription analysis of immortalized porcine intestinal epithelial cell clone J2 (IPEC-J2) cells after PEDV infection, we found that the mRNA expression of the PABPC1 gene significantly up-related at 12–18 h post infection (hpi) [29]. Based on the results, we hypothesize that the PABPC1 gene is involved in the PEDV replication process. In this study, we show that PABPC1 inhibited PEDV replication by interacting with PEDV N protein, which demonstrates a new function of PABPC1 to PEDV infection and enriches the knowledges of interaction between PEDV and host.

## 2. Materials and Methods

### 2.1. Cells and Viruses

Vero cells and 293T cells were grown and maintained in Dulbecco minimum Eagle’s essential medium (DMEM, Gibco, Shanghai, China), supplemented with 10% heat-inactivated fetal bovine serum (FBS, Gibco, Shanghai, China). The cells were cultured at 37 °C in 5% CO_2_. PEDV strain GDS01 (GII subtype, GenBank accession number: KM089829.1) was cultured at 0.1 multiplicity of infection (MOI) and titrated in Vero cells in the presence of trypsin (10 μg/mL). The cells were harvested when 90% of the cells showed the cytopathic effect (CPE) and then subjected to three freeze–thaw cycles. After centrifugation at 10,000× *g* for 10 min at 4 °C, the supernatants were collected for further propagation or stored at −80 °C.

### 2.2. Construction of Expression Plasmids

The gene of PABPC1 (GenBank accession number: XM_007465734.1) was amplified from the genome of IPEC-J2 cells, and the *N* gene of PEDV (GenBank accession number: KM089829.1) was amplified from the genome of GDS01. The amplified genes were cloned into pcDNA3.1(+) vector with FLAG-tag or HA-tag respectively. The PCR primers used in this study were listed as follows: PABPC1-F: 5′-GCCACCATGGAGGCTCCCACCGGGGCT-3′, PABPC1-R: 5′-CCGCTCGAGCGGTTAAACAGTTGGAACTCCAGTGGC-3′; PEDV-*N*-F: 5′-GGGGTACCGCCACCATGGCTTCTGTCAGTTTT-3′, and PEDV-*N*-R: 5′-AAGGAAAAAAGCGGCCGCATTTCCTGTATCGAA-3′. The plasmids were extracted with an E.Z.N.A. Endo-Free Plasmid Mini Kit I (OMEGA, Guangzhou, China) following the manufacturer’s instructions.

### 2.3. Detection of the Antiviral Effect of PABPC1 by Overexpression and siRNA Interference in Vero Cells

Upon reaching 80–90% confluence, Vero cells were transfected with recombinant plasmid pcDNA3.1(+)-PABPC1 with FLAG-tag or empty plasmid using Lipofectamine 3000 according to the manufacturer’s recommendations (Thermo, Shanghai, China). Then, 24 h after transfection, the cells were infected with PEDV at an MOI of 0.1.

To knockdown the endogenous expression of PABPC1, small interfering RNA (siRNA) was introduced. Upon reaching 50% confluence, Vero cells were transfected with PABPC1-specific siRNA (siP1, siP2 and siP3) or negative control siRNA (siNC) twice at 24h intervals. Then, 24 h after 2nd transfection, the cells were infected with PEDV at an MOI of 0.1. The PABPC1-specific siRNA sequences were listed as follows: siP1- 5′-GCCTTAAGTGTGAAAGTAA-3′, siP2- 5′-AGCCACTAAAGCAGTTACA-3′, and siP3- 5‘-ACCTCACTAACCAGTATAT-3′.

The mRNA expression of PEDV *N* and PABPC1 genes was detected by RT-qPCR. The protein expression of PEDV N and PABPC1 was analyzed by Western blot with mouse anti-PEDV polyclonal antibody (prepared by our lab) and mouse anti-FLAG monoclonal antibody (Dia-an, CN). The PEDV titers in the supernatant were detected by plaque assay.

### 2.4. Real-Time Quantitative Reverse Transcription PCR

Total RNA was extracted from Vero cells using TRIzol reagent, and reverse transcribed using a ReverTra Ace qPCR RT Master Mix with gRNA Remover Reagent Kit (TOYOBO, Shanghai, China) in accordance with the manufacturer’s instructions. The reaction systems were configured using Hieff UNICON^®^ Universal Blue qPCR SYBR Green Master Mix (Yea-sen, Shanghai, China). The real-time PCR was performed using a Light-Cycler 480 PCR system (Roche, Basel, Switzerland). The transcript levels of target genes were relatively quantified using the 2^−^^△△CT^ method. The GAPDH gene served as a reference gene. The relative quantity of target mRNA was normalized to that of GAPDH mRNA in the same sample. The specific primers of PABPC1, PEDV *N* and GAPDH were listed as follows: PABPC1-qpcr-F: 5′-TCCAAGAAGGAACCAAGAGACC-3′, PABPC1-qpcr-R: 5′-CGCAGAGGGACAAAAATCAAC-3′, PEDV-*N*-qpcr-F: 5′-GGGTATTGGAGAAAATCCTGATAG-3′, PEDV-*N*-qpcr-R: 5′-AACTGGCGATCTGAGCATAG-3′, GAPDH-qpcr- F: 5′-TACATGTTCCAGTATGATTCCACC-3′, GAPDH-qpcr-R: 5′-CAGTGGACTCCACAACATACGTAG-3′.

### 2.5. Western Blot Assay

The collected cells were efficiently lysed by RIPA Lysis (Beyotime, Shanghai, China) and the proteins were extracted with Extraction Buffer (Beyotime, Shanghai, China) in accordance with the manufacturer’s instructions. The protease inhibitor, phenylmethanesulfonyl fluoride (PMSF), was added to block the endogenous proteolysis. Samples were separated by sodium dodecyl sulfate polyacrylamide gel electrophoresis (SDS-PAGE) and transferred onto a polyvinylidene fluoride (PVDF) membrane. Nonspecific reaction was blocked with 5% skimmed milk in Tris-buffered saline Tween (TBST) buffer. Anti-PABPC1 rabbit monoclonal antibody (Abcam, Cambridge, UK) and anti-GAPDH mouse monoclonal antibody (Proteintech, Rosemont, IL, USA) were used as primary antibodies, respectively, at a ratio of 1:2000. After washing three times with TBST buffer, the membranes were incubated with HRP-conjugated secondary antibody (Proteintech, Rosemont, IL, USA) at a ratio of 1:5000. Protein expression was detected with the commercial ECL kit (Thermo, Waltham, MA, USA), and analyzed with IMAGE J software (v1.8.0, Bethesda, MD, USA).

### 2.6. Plaque Assay

Vero cells were harvested at 9 h post PEDV infection. After three freeze–thaw cycles, the cells were centrifuged at 10,000× *g* for 10 min at 4 °C, and the supernatants were harvested. Plaque assay was performed using Vero cells in 6-well plates when cells reached complete confluence. The virus samples were subjected to 10-fold gradient dilution. Then, the diluted samples were inoculated into Vero cells and discarded after 1 h. The cells were washed three times with phosphate-buffered saline (PBS), and then incubated with 2 mL DMEM medium containing 1% agarose. Finally, the cells were stained with 0.03% neutral red and the virus titers were calculated as plaque-forming units (pfu) /mL.

### 2.7. Mass Spectrometry Analysis and Immunoprecipitation (IP) Assay

Vero cells transfected with pcDNA3.1(+)-PABPC1 plasmid with FLAG-tag were infected with PEDV at an MOI of 0.1 and harvested at 6 hpi. The total proteins were extracted as described above. The supernatants were incubated with anti-FLAG mouse monoclonal antibody for 12 h at 4 °C, then Agarose A+G beads (Beyotime, Shanghai, China) were added. After 6 h incubation, the beads were collected by centrifugation at 2500× *g* for 5 min and washed eight times with cold protein extraction buffer. The beads were boiled in 5× SDS loading buffer to elute bound proteins for mass spectrometry analysis.

To assay the interaction between PABPC1 and PEDV N protein, 293T cells were transfected with pcDNA3.1(+)-PABPC1 or empty plasmid with FLAG-tag and pcDNA3.1(+)-PEDV-N or empty plasmid with HA-tag, respectively. After 24 h, the cells were lysed and incubated with anti-FLAG monoclonal antibody, and the eluted samples were analyzed with anti-FLAG, anti-HA, or anti-PEDV antibodies.

### 2.8. Confocal Microscopy

293T cells grown on a cover glass in a 12-well plate were transfected with pcDNA3.1(+)-PABPC1 or empty plasmid with FLAG-tag and pcDNA3.1(+)-PEDV-N or empty plasmid with HA-tag, respectively. After 24 h, the cells were permeabilized with 0.5% Triton X-100 at room temperature for 15 min and blocked with 5% bovine serum albumin (BSA) in PBST at room temperature for 1 h. The cells were incubated with rabbit anti-FLAG monoclonal antibody (Sigma, Saint Louis, MO, USA) and mouse anti-HA monoclonal antibody (Sigma, Saint Louis, MO, USA), respectively, overnight at 4 °C. After washing three times, the cells were incubated with goat anti-rabbit IgG antibody conjugated to Alexa Fluor 594 and goat anti-mouse IgG antibody conjugated to Alexa Fluor 647 (Sigma, Saint Louis, MO, USA), respectively. Fluorescent images were acquired using the Leica TCS SP5 confocal microscope (Leica, Wetzlar, Germany).

### 2.9. Statistical Analysis

Each experiment was repeated at least three times. Values were expressed as mean ± SD. Statistical analysis were performed using GraphPad Prism5 software (v5.0, San Diego, CA, USA) with Student’s *t*-test. A *p* value < 0.5 was considered statistically significant and labeled as an asterisk in the figures. *, *p* < 0.05; **, *p* < 0.01; ***, *p* < 0.001.

## 3. Results

### 3.1. PEDV Infection First Upregulates and then Downregulates the Expression of PABPC1 in Vero Cells

**Figure 1:**
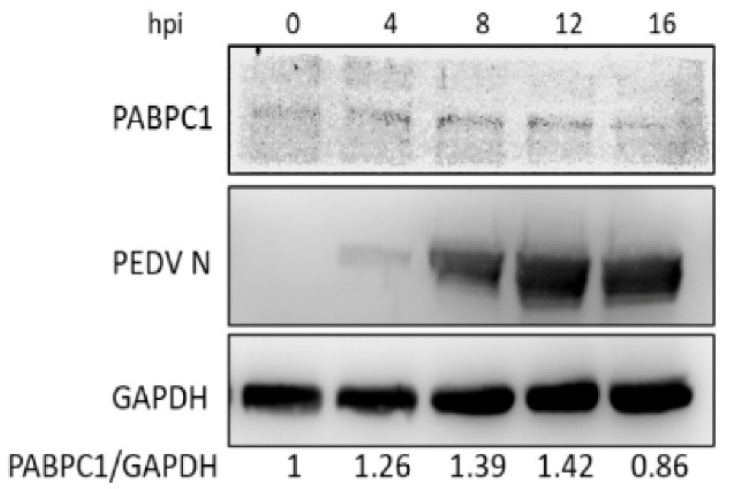
Expression changes in PABPC1 at protein level in Vero cells after PEDV infection. The bottom numbers mean the relative expression folds of PABPC1 protein analyzed by grayscale scanning.

Many cell proteins have been identified as participating in host antiviral activities after PEDV infection in vivo or in vitro [12,13,14,30,31]. The present study focused on whether PABPC1 was involved in PEDV infection process. The changes in PABPC1 protein expression in Vero cells were measured after PEDV infection at an MOI of 0.1. As shown in Figure 1, the protein expression of PABPC1 was upregulated at 8 hpi, reached the highest level at 12 hpi, and downregulated at 16 hpi (Figure 1). These results suggest that, after PEDV infection, the expression of PABPC1 was upregulated at the early infection stage and downregulated at the later infection stage.

### 3.2. Overexpression of PABPC1 Inhibits PEDV Replication in Vero Cells

**Figure 2:**
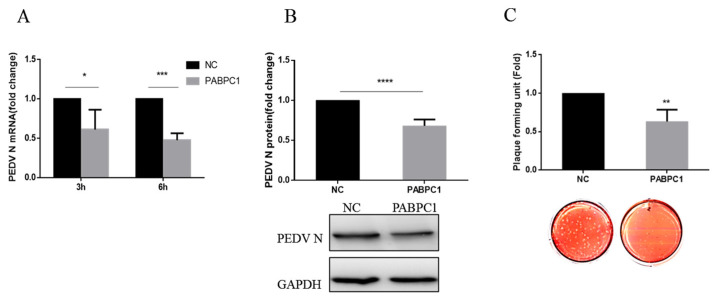
Overexpression of PABPC1 inhibits PEDV replication in Vero cells. (**A**) The mRNA expression changes in *N* gene after the overexpression of PABPC1. (**B**) Changes in N-protein expression after the overexpression of PABPC1. (**C**) Changes in PEDV titers after the overexpression of PABPC1. Values represent means ± SD from three independent experiments. * means *p* < 0.05; ** means *p* < 0.01; *** means *p* < 0.001, **** means *p* < 0.0001.

To explore whether PABPC1 could affect PEDV infection, we examined PEDV N-protein expression level and virus titers after PABPC1 was overexpressed. Vero cells were transfected with pcDNA3.1(+)-PABPC1 recombinant plasmid or empty plasmid for 24 h, then the cells were infected with PEDV at an MOI of 0.1. The cellular RNA was extracted to detect PEDV *N*-gene expression level by RT-qPCR at 3 hpi and 6 hpi. The PABPC1 and PEDV N-protein expression levels were confirmed by Western blot at 6 hpi. The cell-culture supernatants were collected to determine the virus titers by plaque assay at 9 hpi. Compared with the negative control, the mRNA expression level of *N* gene was significantly decreased in PABPC1-overexpressed cells (Figure 2A), and the expression level of *N* protein (Figure 2B) and virus titers (Figure 2C) showed a significant decrease as well. These data suggested that the overexpression of PABPC1 inhibited PEDV replication.

### 3.3. Knockdown of PABPC1 Expression Promotes PEDV Replication in Vero Cells

**Figure 3:**
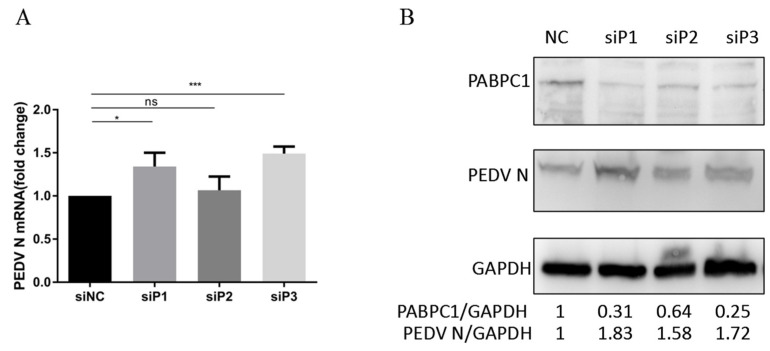
Knockdown of PABPC1 expression by siRNA promotes PEDV replication in Vero cells. (**A**) The changes in *N*-gene expression after the knockdown of PABPC1 by siRNA. (**B**) The changes in N-protein expression after the knockdown of PABPC1 by siRNA. * means *p* < 0.05; *** means *p* < 0.001, ns means *p* > 0.05.

To further clarify the role of PABPC1 in PEDV replication, we knocked-down the endogenous expression of PABPC1 with specific siRNA in Vero cells. Western blot assay confirmed all three synthesized siRNA targeting PABPC1 decreased the expression of endogenous PABPC1 (Figure 3B). At 24 h after siRNA transfection, Vero cells were infected with PEDV at a MOI of 0.1. At 6 hpi, the mRNA expression level of *N* gene was detected by RT-qPCR, and the expression level of N protein was confirmed by Western blot analysis. Compared with negative control, the mRNA level of *N* gene showed significant upregulation in siP1- and siP3-transfected cells (Figure 3A). The expression level of N protein increased by 1.83, 1.58, and 1.72 times in siP1, siP2, and siP3-transfected cells respectively (Figure 3B). Overall, these results proved that the knockdown of PABPC1 expression promoted PEDV replication.

### 3.4. PABPC1 Interacts Directly with N Protein of PEDV

**Figure 4:**
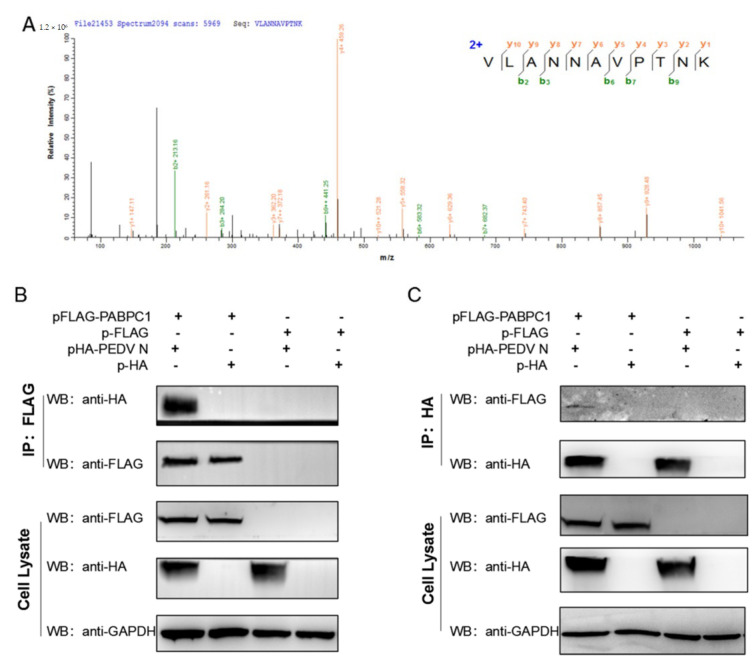
PABPC1 interacts directly with PEDV N protein. (**A**) Mass spectrum data of the potential PABPC1-interacting proteins in PEDV-infected Vero cells. (**B**) Co-immunoprecipitation assay with FLAG-tag antibody in 293T cells after transfection with pcDNA3.1(+)-FLAG-PABPC1 and pcDNA3.1(+)-HA-PEDV-N. (**C**) Co-immunoprecipitation assay with HA-tag antibody in 293T cells after transfection with pcDNA3.1(+)-FLAG-PABPC1 and pcDNA3.1(+)-HA-PEDV-N.

**Figure 5:**
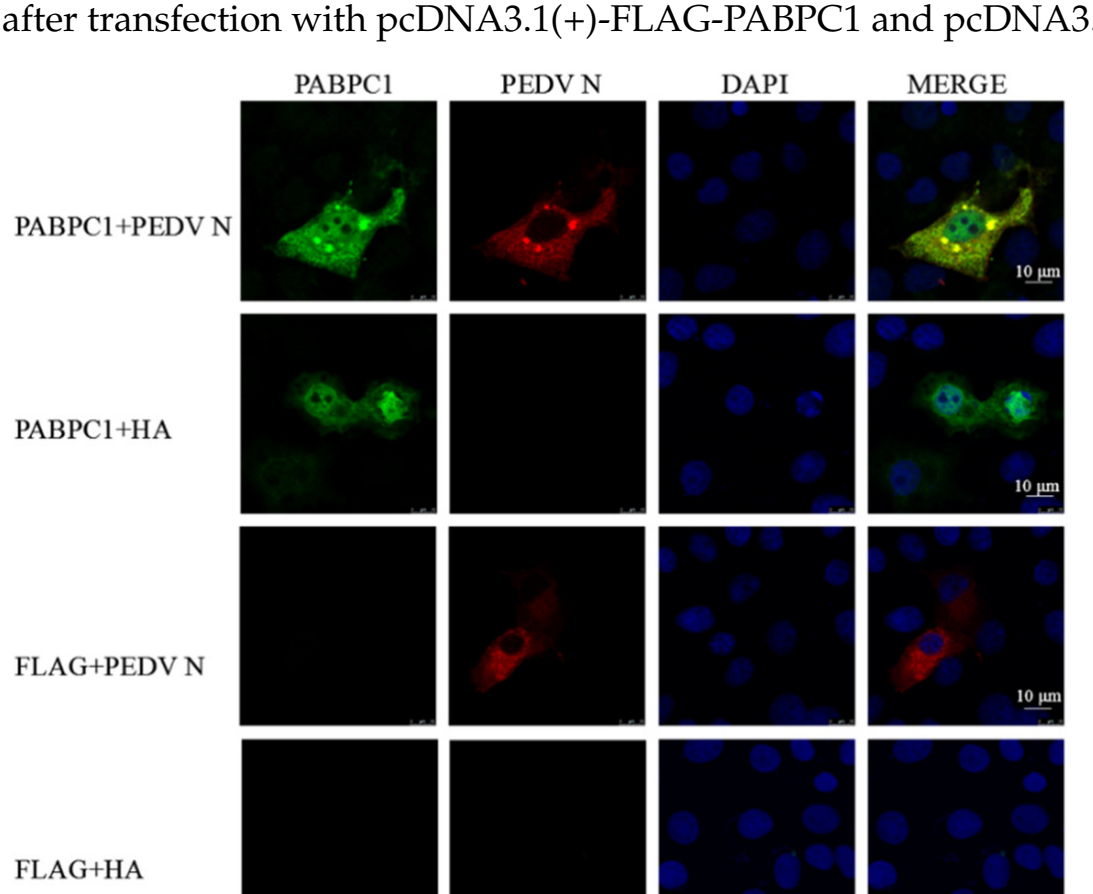
Co-location of PABPC1 and N protein. The scale bars indicate 10 μm.

To further investigate the relationship between PABPC1 and PEDV, the FLAG-tag antibody was used to precipitate the proteins of FLAG-PABPC1-overexpressed and PEDV-infected Vero cells, and the precipitated product was analyzed by mass spectrometry. Multiple proteins, including PEDV N protein, were detected in the precipitated product, indicating that PEDV N protein could interact with PABPC1 in Vero cells (Figure 4A). Thus, we detected the interaction between PABPC1 and PEDV N protein. Firstly, the cellular locations of PABPC1 and N protein were assayed using confocal microscopy by overexpressing them with different tags in 293T cells. Results showed that PABPC1 was mainly distributed in the cytoplasm and there was a small amount of PABPC1 in the nucleus; N protein was mainly distributed in the cytoplasm (Figure 5). There were obvious co-localizations of PABPC1 with N protein in 293T cells. Subsequently, to confirm the interaction, the immunoprecipitation assays were performed by overexpressing PABPC1 and N proteins with different tags in 293T cells. As shown in Figure 4B, N protein with HA-tag can be detected after immunoprecipitation using anti-FLAG monoclonal antibody in 293T cells, and vice versa (Figure 4C). These results confirmed the interaction between PABPC1 and PEDV N protein.

## 4. Discussion

Viruses are non-cellular life forms that must parasitize within cells to proliferate. During virus invasion into host cells, a series of complex interactions occurs between the virus and the host cell, including the regulation and modification of the cell by the virus and antiviral action by the cellular factors. Up to now, several antiviral factors have been explored during PEDV infection, such as bone marrow stromal cell antigen 2, transferrin receptor 1, cholesterol 25-hydroxylase, GTPase-activating protein-binding protein 1, interleukin-11, and IFN-λ [12,13,14,15,16,17,18,19]. The study of host antiviral factors can help to better understand the host–virus interaction. In this study, we explored and identified that the poly(A)-binding protein directly participated in PEDV replication via interaction with N protein.

Coronavirus replication involves not only viral proteins, but also cellular proteins, which are subverted from the normal functions of the host to play roles in the viral replication cycle. Several cellular proteins have been shown to bind to the regulatory elements of mouse hepatitis virus RNA, including the 5′ and 3′ ends of the genomic RNA and the 3′ end of the negative-strand RNA [32,33,34,35,36,37]. PABP is known to interact specifically with poly(A), and the binding of PABP to the 3′-UTR of the defective-interfering (DI) RNA replicons corrects the ability of the DI RNA to replicate, suggesting that the interaction between PABP and the poly(A) tail may affect coronavirus RNA replication [34,38,39,40,41]. However, metazoans often encode multiple cytoplasmic PABPs; which PABPs play a key role in the replication of the coronavirus genome has not yet been studied clearly. Jiao et al. have proved that PABPC4 broadly inhibits coronavirus replication by degrading N protein through selective autophagy [28]. Until now, PABP’s effect on the infection of PEDV remains unclear. In our previous studies about transcription analysis of IPEC-J2 cells after PEDV infection, we found that the mRNA expression of the *PABPC1* gene significantly upregulated at 12–18 hpi [41]. In this study, we identified that PABPC1 protein expression upregulated in the early stage and downregulated at the later infection stage of PEDV infection in Vero cells (Figure 1). Thus, we speculated that PABPC1 is involved in PEDV replication. Then we overexpressed and knocked-down PABPC1 to explore the role of PABPC1 in PEDV replication. After the overexpression of PABPC1, the mRNA and protein expression level of PEDV N, as well as virus titers, were significantly downregulated (Figure 2). After the knock-down of PABPC1 expression by specific siRNA, PEDV replication was promoted with significant upregulated expression of N protein in mRNA and protein level (Figure 3). These results demonstrate that PABPC1 inhibits PEDV replication, and it is the first time the negative effects of PABP in PEDV replication have been reported.

Furthermore, to identify the mechanism of PABPC1 inhibition of PEDV replication, immunoprecipitation assay and mass spectrometry were carried out. In the study of Tsai et al. about the roles of interactions among the poly(A) tail, coronavirus N protein, and PABP in the regulation of coronavirus gene expression, they conclude that N protein competes with PABP to bind to the poly(A) tail, with high affinity, and results in translation inhibition [42]. In our mass spectrometry results, N protein was detected (Figure 4A). Protein co-location and immunoprecipitation assay confirmed the interaction of PABPC1 with N protein in the cytoplasm. As well as N protein, eIF4A, eIF3B and eIF3C proteins were also detected in mass spectrometry, which indicates that these three proteins may be involved in the interaction of PABPC1 and N protein; further study is needed to prove it.

In conclusion, we demonstrated PABPC1, as an antiviral factor, inhibited PEDV replication at both the transcription and translation level by interaction with N protein. This study identified a new function of PABPC1 in PEDV infection and enriched the knowledge of interaction between PEDV and host.
